# A sense of ginger fraud: prevalence and deconstruction of the China-European union supply chain

**DOI:** 10.1038/s41538-022-00166-y

**Published:** 2022-11-04

**Authors:** Qing Han, Sara W. Erasmus, Christopher T. Elliott, Saskia M. van Ruth

**Affiliations:** 1 Food Quality & Design Group, Wageningen University & Research, P.O. Box 17, 6700 AA Wageningen, The Netherlands; 2 Institute for Global Food Security, Biological Sciences, 19 Chlorine Gardens, Queen’s University Belfast, BT9 5DL Belfast, Northern Ireland UK; 3 School of Food Science and Technology, Faculty of Science and Technology, Thammasat University, 99 Mhu 18, Pahonyothin Road, Khong Luang, Pathum Thani 12120 Thailand

**Keywords:** Agriculture, Industry

## Abstract

As an important spice, ginger has been widely distributed in the Chinese and the European Union (EU) markets, the two largest trading areas, in various forms. The ginger supply chain between China and the EU is long and complex, providing opportunities for fraudsters to deceive consumers. However, limited attention has been given to food fraud in ginger, and there is a lack of research on this topic. In this review, ginger was used as an example for interpreting the fraud issues within low-priced and high-trade volume spice products. This review aims to summarize the open access information from food and food fraud databases, literature, and stakeholders about ginger fraud, and to map, deconstruct and analyse the food fraud vulnerability in the supply chain. In addition, potential testing strategies to detect ginger fraud were also discussed. The investigation of food fraud databases, a semi-structured literature review and online interviews with stakeholders revealed that adulteration is the major fraud type in ginger products. And the most vulnerable ginger products are ground ginger and finely processed ginger. The ginger supply chain from China to the EU comprises nine stages and is medium vulnerable to food fraud, both in regard to opportunities and motivational drivers. To ensure the integrity of the ginger supply chain, there is a need to apply fraud vulnerability tools in the companies of the industry. In addition, screening and confirmatory techniques based on the characteristics of ginger should be utilised for monitoring fraud issues in the supply chain.

## Introduction

Food fraud is generally defined as one of the behaviours that deceive consumers for financial gain^1^. Food fraud types include but are not limited to, adulteration, substitution, addition, tampering or misrepresentation of food, food ingredients or food packing; or false and/or misleading statements made about a product^2^. Food fraud has troubled the food industry throughout history^3^. The 2008 melamine-contaminated milk powder scandal in China and the 2013 horsemeat scandal in the European Union (EU) shed light on the problem again and highlighted that food fraud is a vital topic that has been greatly overlooked by the food industry for many years^4,5^. Food fraud can cause economic and reputational damages to food companies and the associated industry, in addition, some fraud incidents have resulted in serious public health risks such as allergies and poisoning^6,7^. The reported food fraud scandals not only caused significant financial losses to the food industry and brought about food safety or public risk events but also reduced consumers’ confidence in brands.

The spice industry is one of the top three industries prone to food fraud^8^. Although only consumed in small quantities, spices play a significant role in the food industry as it is an important ingredient in a huge variety of foods and beverages. Its unique position in the food industry, complex supply chain and high price by weight make spice highly vulnerable to food fraud^9^. In addition, the time it takes from harvest to the consumption of spice can be years; further decreasing the transparency of the spice supply chain^10^. Nowadays, more research is being conducted on food fraud issues in the spice industry, especially on developing analytical techniques for spice authentication^11^. Nevertheless, limited research has focused on summarizing the fraud issues of a specific spice, as well as the intricacies of spice supply chain networks and the fraud risk factors hidden therein. In addition, low-priced and high trade volume spices receive limited attention compared with expensive spices such as saffron and vanilla. In 2018, a review by Galvin-King et al.^12^ gave a general insight into the spice supply chain and looked at the type and effect of fraud in spices and herbs. This review created a need for further research on the topic; especially focused on specific spices.

From a global perspective, the EU is one of the largest consumption areas of spice^13^. Nearly 95% of the EU’s imports of spices come from developing countries, while China is the EU’s largest spice trading partner that mainly supplies ginger and capsicums^14^. Among all spices circulated worldwide, ginger is a representative of the low-priced and high trade volume spice products because of its mass trade volume and wide application in foods. Ginger is one of the most common spices used for flavouring dishes in China, and it is also one of the most important spices exported to the European market because of the huge import volumes^14,15^. In 2018, the import volume of ginger (in all forms) was 127,259 tonnes, ranking first in the import of spices and herbs from developing countries, moreover, China accounted for almost 45% of ginger supplies from developing countries to the EU^16^.

However, even though ginger, especially ground ginger, has been labelled at high risk of food fraud according to the Food Fraud Risk Information^17^, fraud issues in ginger products have not been investigated and summarized. The overall situation regarding ginger fraud remains unclear. Moreover, the current understanding of the ginger supply chain from China to the EU is still the general impression of the traditional system of the spice supply chain^18^. The hidden issues in the opaque ginger supply chain limit the ability of stakeholders to guarantee the authenticity of this important spice. Therefore, it is necessary to provide a general summary of food fraud issues in ginger products and to map, deconstruct and analyse the supply chain based on the current information. This paper aims to summarize the open access data in the food fraud databases and the literature about ginger fraud, comprehend the structure of the China-EU ginger supply chain to identify the most vulnerable nodes and propose potential technical solutions that can be used to mitigate ginger fraud. This review can be used as an example for the spice industry as a solution to the fraud issue in many other low-price high trade volume spices.

## Methods

An investigation of food fraud databases, a semi-structured literature search, online interviews with key stakeholders and a food fraud vulnerability assessment was performed to identify ginger products with fraud concerns, explore associated fraud issues, and map the ginger supply chain between China and the EU.

### Investigation of food fraud databases

The databases, as described in Supplementary Table 1 related to food and food fraud cases were investigated to summarize the information about ginger and ginger fraud issues. The specific keyword, “ginger”, was used to search for information related to ginger and ginger fraud to ensure all types of ginger products were included while searching. The information generated from the Food and Agriculture Organization Corporate Statistical Database (FAOSTAT) and Tridge were about the latest price, production, export and import volume of ginger in China and the EU. The Food and Feed Safety Alerts Portal (RASFF Portal), Food Fraud Risk Information, Decernis Food Fraud Database, Food Adulteration Incidents Registry, Recalls, Market Withdrawals, and Safety Alerts and Medical Information System (MEDISYS) were screened for ginger fraud issues covering all fraud types and all publication years. A total of 32 cases of ginger fraud were found in the food fraud databases and summarized by product types, databases, fraud types and detailed issues.

### Semi-structured literature search

For the semi-structured literature search, Web of Science, Scopus, and Google Scholar were used as databases to source relevant articles written in the English language, covering all publication years up to 2021. During the process, specific keywords, and the combination of these keywords with Boolean operators were included. For the search strategy to obtain information related to ginger supply chains, the following search string was used within all fields of the databases: “spice” OR “ginger” AND “supply chain” OR “supply chain network” AND “map” OR “diagram” OR “model”. For the search strategy of fraud issues related to ginger, the following search string was used within all fields of the databases: “ginger” AND “fraud” OR “adulteration” OR “unapproved processing” OR “undeclared processing” OR “mislabelling” OR “misrepresentation”.

The above-mentioned keywords of ginger fraud were selected from the “CWA 17369: Authentic and fraud in the feed and food chain – Concepts, terms and definitions” standard published by the European Committee for Standardization and the detailed definition of the keywords are as fellow: adulteration is the intentional addition of inferior materials in foods, even non-food adulterants, to increase profit margins; misrepresentation/mislabelling refer to when the label is not in accordance by the actual food product characteristics; unapproved/undeclared processing is intentionally improving perceived quality or covering the deficiencies of food products^19,20^. During the search processing, the inclusion and exclusion criteria (Table 1) were used to determine if the articles were pertaining to the primary research aims. Governmental websites were also used to search for ginger supply chain and ginger fraud issues using the above-mentioned keywords. A total of 13 relevant articles including five government papers were found and formed the basis of the ginger supply chains data. The references of 13 relevant articles can be found in Supplementary Table 2. A total of 31 relevant articles related to ginger fraud that fit the inclusion and exclusion criteria were found and summarized.

**Table 1:** Inclusion and exclusion criteria for the review.

Inclusion criteria	Exclusion criteria
Food Science field	Non-Food Science field i.e., medical science
English language	Non-English language
Ginger or ginger products and food fraud or at least one type of food fraud must be mentioned together	Related products only described ginger as the product flavour
	Studies examining food safety with no food fraud component will be excluded
	Duplicate study

### Validation through online interviews with supply chain stakeholders

Online interviews with stakeholders were conducted to validate the accuracy of the information obtained from the literature and the food fraud databases. In addition, the information acquired during the interviews were used as supplementary information for the results generated from the investigation of food fraud databases and semi-structured literature search. This step aims to eliminate the potential cognition gaps between the real industry, researchers (literature) and organizations/authorities (food fraud databases). A total of five stakeholders (i.e., three processors, one exporter and one trader) from three actor groups in the ginger supply chain were requested to participate in a 30-min online interview. All interviewees’ positions were quality assurance managers, two from China and three from the EU. The interviews were conducted online in English and Chinese, depending on the nationality of the interviewees, in three steps. First, the aim and major content of the interview were explained. Second, the interviewees were requested to introduce themselves and basic information about their companies. Third, the interviewees were requested to answer questions about their ginger products, supply chains and potential fraud risk factors. The full interview questions that were used in step three are shown in Supplementary Table 3. The interviewees and their affiliated companies were treated anonymously, while all original materials used in the interview process were treated confidentially and would not be published in any form. The interviews served as validation and supplementation. Therefore, the collected information is presented as a complement along with inferences drawn from the food fraud databases and the literature as a whole.

### Vulnerability assessment of the ginger supply chain from China to the EU

After mapping the ginger supply chain based on the information obtained from semi-structured literature search and online interview with supply chain stakeholders, the food fraud vulnerability of the supply chain was assessed to gain a better understanding of the supply chain from a fraudulent perspective. The food fraud vulnerability of the ginger supply chain from China to the EU was determined by using the free online food fraud vulnerability assessment tool, developed by the non-profit SSAFE organization in partnership with Wageningen University, VU University Amsterdam^1^. This is a science-based food fraud vulnerability self-assessment questionnaire consisting of 50 questions to evaluate the three key elements of food fraud vulnerability: Opportunities, Motivations and Control measures. Each question has three optional answers describing low, medium, and high vulnerability situations of the associated indicator. For the indicators from Opportunities and Motivations, answers with scores 1, 2 and 3 reflected a low, medium, and high vulnerability level, respectively. For the indicators from Control measures, the answers with the scores 1, 2 and 3 reflected a high, medium, and low vulnerability, respectively. The questions used in this study were pre-selected and slightly modified based on the available information acquired from food fraud databases, semi-structured literature search and online interview with stakeholders before being further used. The results were determined by the highest frequency of perceived vulnerability for all stages/indicators/elements. The frequency was determined by the following Eq. (1):

1 $$Fi = \frac{{X{{{\mathrm{ij}}}}}}{{Yj}}$$

Where *Fi* is the frequency of score *i* (*i* = 1, 2, 3), *Xij* is the number of observations which get the score *i* in the *j* stages/indicators/elements, *Yj* is the total number of the observation in the *j* stages/indicators/elements.

## Fraud prevalence in ginger

### Ginger products with fraud concerns

Ginger (*Zingiber officinale*) is one of the most commonly used spices^21^. Because of its unique flavour and potential health benefits to the human body and brain, ginger is widely used as a spice and traditional medicine^22^. The unique flavour of ginger makes it suitable to be used for various food products such as vegetables, confectionery, soft and alcoholic beverages, pickles, and biscuits. Ginger can be used in numerous forms, such as fresh, dried, grounded, pickled, preserved, and crystallized. The harvest time of ginger rhizomes depends on their intended use. For fresh, preserved, or pickled ginger, young and immature ginger is primarily used as it is juicy, with a mild taste and has a thin skin^23^. The content of fibre and volatile organic compounds of ginger continues to increase with age^24^. Therefore, for dried ginger, it is best to use mature rhizomes, which have a sufficient aroma, flavour and pungency^25^. In addition, the ideal type of ginger for cooking is ginger harvested eight to nine months after planting because the content of volatile organic compounds and pungent compounds reach a maximum of about nine months after planting^26^. In this section, the ginger products with food fraud concerns were summarized from the literature and food fraud databases and classified according to the degree of processing (Table 2).

**Table 2:** Ginger products with food fraud concerns.

Products	Degree of processing	Description
Fresh ginger	n.a	Fresh ginger is the young and immature ginger rhizome that is commonly used as a spice in cooking^16^.
Dried ginger	Whole form	Dried ginger is obtained by air-drying or mechanical drying. Dried ginger is commonly used in spices, masalas, curries, and stews. According to the Quality Minima Document published by the European Spice Association (ESA), the moisture content of dried ginger should not exceed 12%^74^.
	Sliced	After peeling, the fresh ginger rhizome is thinly sliced and dried out.
	Grounded	Ground ginger is made by drying out peeled fresh ginger rhizome, then grinding it to a fine powder. Ground ginger is a key ingredient of many ginger-related foods in the food processing industry, including spices mixture producers, meat processing industry, sauces and condiments industry, convenience food and snacks and the drinks and beverages industry^16^.
Finely processed ginger	Preserved ginger	Preserved ginger is made from fresh ginger that has been peeled or sliced, then cooked in and preserved in a sugar syrup^26^. Commonly, preserved ginger is used in desserts.
	Pickled ginger	Pickled ginger is thinly sliced ginger with a sweet and sour flavour, which is obtained by marinating in a sugar and vinegar solution. It is made from young ginger and is usually served with sushi. Pickled ginger typically has a pale yellow to slight appearance during the pickling process.
	Crystallized ginger	Crystallized ginger is also known as candied ginger. Fresh ginger is slowly cooked in sugar water and then tumbled in coarse sugar to preserve. Crystallized ginger has a sweet and spicy taste, and it is usually served as dessert.
	Ginger oil	Ginger oil is an essential oil obtained by steam distillation of the rhizome of ginger. It has the aroma and flavour of ginger but lacks the pungency. The main application of ginger oil in food industry is the flavouring of beverages and confectionery^75^.

### Fraud prevalence reported in the food fraud databases and the literature

#### Fraud prevalence reported in the food fraud databases

The search results from the food fraud databases in Table 3 indicate that the amount of information in the different databases was uneven. Food fraud issues in ginger products could only be found in three of five databases: 20 reports in the RASFF portal, two reports in Food Fraud Risk Information and 10 reports in the Decernis Food Fraud Database. Almost all information in the databases was related to adulteration and unapproved processing or undeclared processing, except one entry about the misrepresentation of geographical origin listed in the Food Fraud Risk Information and two cases about the misrepresentation of production system claims in the Decernis Food Fraud Database. In addition, processed ginger products, such as pickled ginger, preserved ginger, and sliced ginger, were the most common products recorded in the food fraud databases. In the RASFF portal, the type of ginger products, their adulterants and notifying countries were described. The information from the Decernis Food Fraud Database is more specific where the reason for adulteration, date, location, and reference can be found. Although ginger has been labelled at high risk of food fraud in the Food Fraud Risk Information, there is only a statement and no detailed information and case studies mentioned to substantiate the risk assignment.

**Table 3:** The search results of the different food fraud databases.

Databases	Products	Fraud types	Detailed issues	Number of cases
Food And Feed Safety Alerts Portal (RASFF Portal)	Pickled ginger; Sliced ginger; Candied ginger in syrup; Minced ginger; Preserved ginger; Ginger paste	Adulteration	Adulterants: Preservative; Sweetener	12
		Unapproved/ undeclared processing	Colorant	8
Decernis Food Fraud Database	Ginger; Ground ginger; Ginger paste; Ginger oil	Adulteration	Adulterants: Bean powder; Ultramarine blue; Lead; Mashed potatoes; Acid; Onion; Banana pulp; Soybean oil	8
		Misrepresentation/ mislabelling	Misrepresentation of production system claims	2
Food Fraud Risk Information	Ginger; Powdered ginger	Adulteration	Adulterants: cheap fillers	1
		Misrepresentation/ mislabelling	Misrepresentation of geographical origin	1
Food Adulteration Incidents Registry				None
Recalls, Market Withdrawals, & Safety Alerts				None
Medical Information System (MEDISYS)				None

Note. The detailed information obtained from the databases are shown in Supplementary Table 4 (provided as Supplementary material).

#### Fraud prevalence reported in the literature

The search results from the Web of Science, Scopus and Google Scholar (as shown in Table 4) indicates that most of the literature mentioned ginger adulteration, while some articles also gave examples of adulterants^27–29^, however, only a few articles directly focussed on the description or the detection of ginger fraud and the type of ginger products was also not described in detail in the articles^30,31^. In most cases, ginger adulteration is only used as an example in other food fraud studies^12,27^. In addition, misrepresentation/mislabelling of ginger and unapproved/undeclared processing of ginger were less common in the literature, only three out of 31 studies indicated these fraud issues^31–33^.

**Table 4:** The search results of ginger fraud from the literature.

Keywords	Products	Detailed issues	Number of publications
Web of Science			
“ginger” AND “fraud”			None
“ginger” AND “adulteration”	Ginger; Ginger oil	Adulterants: not mentioned	6
“ginger” AND “unapproved processing” OR “undeclared processing”	Ginger	Sulfur	1
“ginger” AND “mislabeling” OR “misrepresentation”			None
Scopus			
“ginger” AND “fraud”	Ginger	Adulterants: not mentioned	1
“ginger” AND “adulteration”	Overlapped with Web of science		
“ginger” AND “unapproved processing” OR “undeclared processing”	Overlapped with Web of science		
“ginger” AND “mislabeling” OR “misrepresentation”	Overlapped with Web of science		
Google Scholar			
“ginger” AND “fraud”			None
“ginger” AND “adulteration”	Ginger; Ground ginger	Adulterants: Spent ginger; Chili; Capsicum; Galangal; Starch; Extraneous mineral matter; Cornstarch; Bran; Calcium hydroxide; Turmeric powder; Wheat flour	23
“ginger” AND “unapproved processing” OR “undeclared processing”			None
“ginger” AND “mislabeling” OR “misrepresentation”	Ginger	Misrepresentation of geographical origin	2

Note. All references of the articles are listed as supplementary material in Supplementary Table 5.

#### Summary and comparison of the two information sources

The search results of the databases and the literature led to the same conclusions, adulteration, misrepresentation/mislabelling and unapproved/undeclared processing appeared in two information sources. Yet the details of information related to the adulteration of ginger products were quite different as can be seen in Table 5. Adulteration of ginger products is the most crucial problem in the ginger industry which has been mentioned 50 out of 65 times in all results. In the food fraud databases, incidences of adulteration were reported as the addition of unapproved food additives (undeclared sweetener and undeclared preservative) to improve the flavour and shelf life of processed ginger^34^. However, in the literature results, the adulteration of ginger was related to the addition of other powdered materials to increase the bulk weight of ground ginger^35^. The search results from food fraud databases and literature showed that the unapproved processing of ginger involved the use of sulphur smoke and the addition of colorants which were mentioned nine out of 65 times in all results. For both sources, misrepresentation/mislabelling of ginger included fraudulent geographical origin and production system claims (only mentioned five out of 65 times in all results). According to the answers from the interviewees, it is the industry’s consensus that ground ginger is more vulnerable to food fraud, especially by adulteration with foreign material. This is consistent with the findings from the literature search results.

**Table 5:** The summarization of the fraud prevalence from two sources.

Fraud prevalence in ginger products		Food fraud databases	Literature
Number of reports (In total 65)		32	33
Fraud type	Adulteration	21	29
	Misrepresentation/mislabelling	3	2
	Unapproved/undeclared processing	8	1
Discrepancy between the search results		Main targeted product	
		Processed ginger	Ground ginger
		Addition of unapproved food additives to improve the flavour and shelf life of processed ginger	Addition of other materials to increase the bulk weight of ground ginger

The results of the food fraud databases showed that food fraud in ginger, a low-priced and high-trade volume spice product, is not only an example in the literature but a real-world issue. The discrepancy between the search results of the food fraud database and the literature may be either because there is less adulterated ground ginger in the actual market compared with fine processed ginger, or the limited technology available together with no/restricted market monitoring means that the adulteration of ground ginger remains hidden. These two possible reasons may have contributed to the limited reports of adulterated ground ginger in the food fraud databases, in contrast to the high proportion (but undetailed information) of adulterated ground ginger in all literature.

## The ginger supply chain network: from China to the EU

The complexity and transparency of a supply chain or indeed a network is an important characteristic that helps to determine where fraud risks exist. Therefore, it is pivotal to map and deconstruct the Chinese and the EU ginger market and to identify critical nodes along the chain.

The information about the ginger markets in China and the EU was obtained from the Food and Agriculture Organization Corporate Statistical Database (FAOSTAT) as well as Tridge. The basic scheme of the ginger supply network from China to the EU was generated from 13 articles including five government reports (Supplementary Table 1) related to ginger or spice supply chains. The information acquired from interviews, including the role of the companies, their upper and lower actor groups, their knowledge of the ginger supply network and fraud issues, was used to validate the information obtained from the 13 articles and the supply chain actor groups. The actor groups in the supply chain were connected according to the flow of products from one actor group to the next. In addition, the supply chain was divided into different stages to reduce the complexity of the whole network based on the classification method of the “Guidance on authenticity of herbs and apices industry best practice on assessing and protecting culinary fries herbs and spices” released by British Retail Consortium, Food and Drink Federation and Seasoning and Spice Association^36^. The associated fraud issues at each stage of the supply chain were also identified based on this Guidance.

### The ginger market in China and the EU

#### Export market: China

The demand for ginger is growing annually and is expected to increase in the coming years^16^. In 2019, exported ginger (uncrushed or unground) amounted to US$ 849.5 million, and the total export of crushed or ground ginger was US$ 81.5 million^16^. Based on the export value, the top five exporting countries of ginger are China, the Netherlands, Thailand, Peru, and India. China is the main producing and exporting country of ginger and has more than a 50% share of the global export market. China’s ginger exports in 2019 amounted to US$ 508.3 million, with an export volume of 490,500 tones^37^. Moreover, almost 80% of all suppliers from developing countries to the EU are Chinese suppliers. Among all ginger products exported from China, almost 90% are in uncrushed/unground (whole) forms, while only 10% are crushed or ground products^16^. Ginger production in China has been mechanized, which makes it more competitive than any suppliers. Some provinces in China, such as Shandong, Hebei, Liaoning and Fujian, are known as the main origins of ginger^16^.

#### Import market: the EU

In 2018, the import value of ginger worldwide amounted to US$ 826.4 million, with an import volume of 645,700 tonnes^37^. For the EU, most of the ginger is imported from other countries, especially developing countries. In 2018, more than 70% of imported ginger in the EU came from developing countries^16^. The Netherlands is the largest importer and marketer of ginger in the EU, where the import volume of ginger has grown significantly^16^. Germany is the second largest importer of ginger in the EU, with an import volume of 22,600 tonnes in 2018. Nearly 90% of Germany’s ginger product imports come from developing countries^38^. Italy, Spain and France are medium-sized importers of ginger in the EU and their main supplier is China^16^.

### The ginger supply chain from China to the EU

**Fig. 1:**
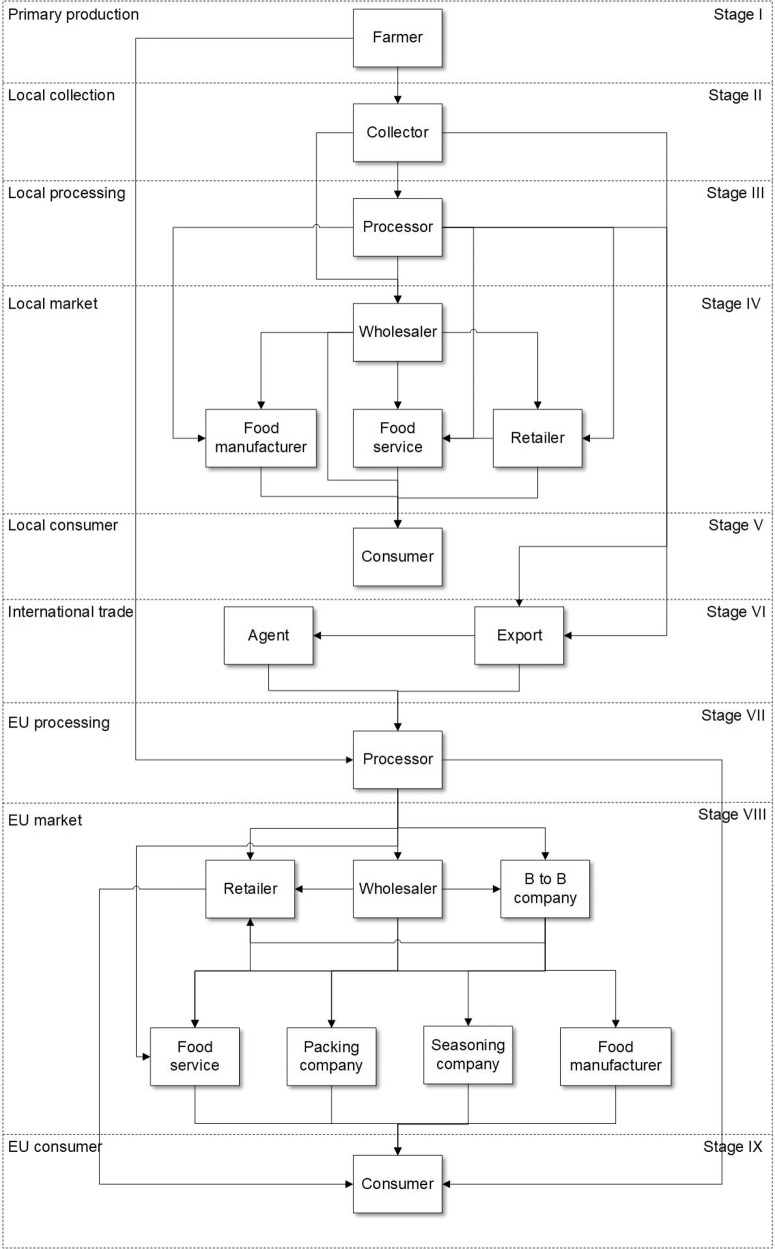
The ginger supply chain from China to the European Union (EU). Note. Each solid small rectangle represents an actor group, each dotted big rectangle represents a stage (from Stage I to Stage IX), and the arrow direction represents the flow of products from one actor group to the next.

The ginger supply network between China and the EU follows a traditional system. The key actor groups in the ginger supply network are presented in Fig. 1. They are farmers, collectors, processors, agents, exporters, wholesalers, retailers, food manufacturers, food service operators, business-to-business (B-to-B) companies, seasoning companies, packaging companies and consumers. From farmers in China to the final consumers in the EU, the ginger supply network can be divided into nine stages according to the classification method of the Guidance on Authenticity of Herbs and Spices^36^. The nine stages are primary production, local collection, local processing, local market, local consumer, international trade, EU processing, EU market and EU consumer. The structure and the food fraud vulnerability of the supply chain were analysed by the stages to gain a better understanding of the supply chain from a fraudulent perspective.

A modification of the SSAFE FFVA tool was applied to assess the fraud vulnerability of each stage and the whole chain with indicators from the tool. The tool was developed as a self-assessment tool for food business operators. However, in the current study the tool was modified to allow a ‘bird’s eye view’ evaluation, i.e. as a third-party tool instead of a tool for a food business operator. The modification of the tool was based on the acquired information from the literature, the databases, and the interviews with stakeholders. The available information from the previous sections includes specific products, detailed fraud issues, potential adulterants, available techniques, the number of historical cases, etc. Therefore, only indicators related to the above-mentioned information were used in this study. Some food fraud factor/indicators of the SSAFE self-assessment FFVA tool focus on the company-specific food business environment. However, these factor/indicators cannot be used at the aggregated level for the ‘bird’s eye’ approach and were omitted in the current study. These inapplicable factor/indicators are listed in Supplementary Table 6. It is important to note that the indicators from the Control measures were all excluded from the assessment as the indicators belonged to the list of inapplicable indicators. The applicable fraud factor/indicators were related to potential threats, represented by food fraud factor/indicators in the Opportunities and Motivations key element groups; therefore, the fraud vulnerability of the ginger supply chain was discussed from two perspectives, opportunities related vulnerability and motivations related vulnerability. The applicable fraud factor/indicators were further divided into the factor/indicators for each stage and the factor/indicators for the whole chain based on the targeting objects of the fraud factor/indicator questions (Table 6 and Table 7). The factor/indicators applied to each stage all belonged to the key element Opportunities including technical opportunities and opportunities in time and space. Whereas the factor/indicators applied to the whole supply chain were from the key elements Opportunities and Motivations. The consequence of the selected ‘bird’s eye approach’ is that the level of vulnerability is determined by the threats in the chain only. In practice these can be mitigated, at least to some extent, by appropriate control measures but this will depend on priorities and capabilities of individual food business operators.

**Table 6:** The fraud factor/indicators targeted on each stage of the supply chain.

Key element	Fraud factor category	Fraud factor/indicator	Questions	Level of vulnerability		
				Answer option1 Low vulnerability	Answer option 2 Medium vulnerability	Answer option 3 High vulnerability
Opportunities	Technical opportunities	Availability of technology and knowledge to commit fraud on final products	1. How available is the technology and knowledge to enable to commit fraud on your final products?	• No technologies and/or adulteration methods are known or available to commit fraud on final products	• Advanced technologies, methods, facilities, and knowledge are required to commit fraud on final products	• Simple/basic technologies and methods are available, and no specialist facilities are required, to commit fraud on final products • The knowledge required for committing fraud on is generally available
		Detectability of food fraud in final products	2. How easily would food fraud of your final products be detected and what kind of methods are available?	• Detection of food fraud of final products is easy and performed with common/simple methods (e.g., visual inspection, smelling)	• Established on-site methods are available for fraud screening (e.g., test kits) but confirmation of food fraud requires additional testing	• Detection and confirmation of food fraud in final products requires advanced laboratory analyses, or testing for food fraud is not available at all
	Opportunities in time and space	Historical evidence of fraud in final products	3. Have fraudulent incidents of similar final products been reported?	• No fraudulent incidents related to any final products are known • No documented evidence/information of fraud is available	• A few fraudulent incidents have occurred with specific final products • Limited documentation and few/no media reports are available	• Many fraudulent incidents have occurred with specific final products • Incidents are well known and documented and have received substantial media attention

**Table 7:** The fraud factor/indicators targeted on the whole supply chain.

Key element	Fraud factor category	Fraud factor/indicator	Questions	Level of vulnerability		
				Answer option1 Low vulnerability	Answer option 2 Medium vulnerability	Answer option 3 High vulnerability
Opportunities	Technical opportunities	The complexity of committing food fraud on raw materials	1. Is it simple or complex to commit food fraud on your raw materials?	• Composition of the materials cannot be modified, and products can only be replaced, i.e., it concerns large objects	• Composition of the raw materials can be modified by mixing with low-quality product-own material or foreign material, i.e., as is feasible with ground products	• Composition of the raw materials can be modified by mixing with low-quality or foreign material and by altering valuable food components
		Availability of technology and knowledge to commit food fraud on raw materials	2. Is the technology and knowledge to commit food fraud on your raw materials generally available?	• Technologies and/or methods to commit food fraud on the raw materials are neither available, known, or reported	• Advanced technologies, methods, facilities, and knowledge are required to commit food fraud on the raw materials	• Simple/basic technologies and methods are available, and no specialist facilities are required, to commit food fraud on the raw materials • The knowledge required for committing food fraud on raw material is generally available
		Detectability of food fraud in raw materials	3. How easily can food fraud in your raw materials be detected and with what kind of methods?	• Detection of food fraud in raw materials is straightforward and performed with common/simple methods (e.g., visual inspection, smelling)	• Established on-site methods are available for fraud screening (e.g., test kits) but confirmation of food fraud requires additional testing	• Detection and confirmation of adulteration of raw materials requires advanced laboratory analysis, or testing for food fraud is not available at all
	Opportunities in time and place	Transparency of the supply chain network	4. How would you describe your part of the food supply chain?	• The supply chain is transparent, with good insight into suppliers and customers • Business relationships are long-term relationships and characterized by trust • The supply chain is integrated and well-coordinated, with comprehensive information exchange across the supply chain	• The supply chain is not fully transparent; only direct suppliers and customer are known • Business relationships are variable; some relationships are long-term, others short-term • Some degree of integration exists across the supply chain; information exchange occurs mainly with direct suppliers and customers	• The supply chain is complex and lacks transparency; typically, customers and suppliers are geographically disbursed • Business relationships are ad-hoc and the price is the main driver for selecting suppliers • No information exchange occurs between direct suppliers and customers
		Historical evidence of fraud in raw materials	5. Have fraudulent incidents of similar raw materials been reported?	• No fraudulent incidents related to raw materials are known • No documented evidence/information of fraud is available	• A few fraudulent incidents have occurred with specific raw materials • Limited documentation and few/no media reports are available	• Many fraudulent incidents have occurred with specific raw materials • Incidents are well known and documented and have received substantial media attention
Motivations	Economic drivers	Economic situation of the raw materials	6. How would you define the supply and pricing of your raw materials?	• Raw materials are readily available • No export bans on raw materials exist • Prices for raw materials are stable • Pricing of raw materials is independent of geographical origin • Prices of substitute raw materials are equivalent	• Stable prices but the supply of raw materials is not readily available • Export bans on raw materials exist in a few countries	• Tight global supplies of raw materials and/or shortages exist • Export bans on raw materials exist in many countries • Price spikes of raw materials are common • Large differences in prices of materials from different geographical regions • Prices of substitute raw materials vary greatly
		Valuable components or attributes of products	7. Do special attributes or components determine the value of your raw materials?	• The value of raw materials is not determined by its composition, way of production or origin	• The value of raw materials is influenced by its composition	• Value of raw materials is greatly determined by its composition, way of production and/or origin
		Corruption level in the country	8. How would you rate the corruption level (according to the Transparency International Corruption Perception Index) in the countries where your company is active? (www.transparency.org/cpi)	• The company is active in countries with low levels of corruption (rated 1–25 on the Index)	• The company is active in countries with medium levels of corruption (rated 26–75 on the Index)	• The company is active in countries with high levels of corruption (rated 76 and above on the Index)
		Economic conditions branch of industry	9. How would you describe the economic health across your sector of the food supply chain (i.e., your company and your direct competitors)?	• The company operates in a growing market(s)	• The company operates in a stable market • The company operates in growing and declining markets	• The company operates in a declining market(s)
		Price asymmetries	10. Are there price differences as a result of regulatory differences across countries?	• The price policy of food ingredients and food products is similar for all countries	• The price policy of food ingredients and food products is different in some countries	• The price policy of food ingredients and food products varies considerably across different countries
	Culture and behaviour	Historical evidence branch of industry	11. How common are criminal offences across your sector of the food supply chain?	• There is no evidence of fraudulent activity or other forms of law-breaking in our sector	• There may have been incidences of fraud across the sector but there is no specific information available	• There is well-known and documented evidence of fraudulent activity across our sector of the food industry

The assessment of the opportunities-related vulnerability of the chain was conducted by accessing the opportunities-related vulnerability of each stage using stage-targeted fraud factor/indicators (Table 6) and combined with the whole chain targeted factor/indicators (Table 7) to have the overall opportunities-related vulnerability of the whole chain. The assessment of motivations-related vulnerability of the chain was conducted using the fraud factor/indicators in Table 7. The opportunities and motivations related vulnerability of the nine stages and the whole chain are described below.

#### Primary production

The primary production stage of the food supply chain includes agricultural activities, aquaculture and other similar processes related to raw food materials^39^. Regarding the ginger supply chain, activities in this stage related to the harvest, handling, and storage of fresh ginger before it moves to either processing or distribution. At this stage, farmers may use acid wash ginger or use sulphur smoke ginger (unapproved/undeclared processing) to improve the appearance of the ginger, as reported on social media^40,41^. Such fraudulent activities do not require advanced technology, methods and/or facilities suggesting the factor/indicator ‘Availability of technology and knowledge to commit food fraud on final products’ is high vulnerability (Table 6 - Question 1 - Answer option 3 - score 3). Unapproved/undeclared processing of ginger is easily identified by the abnormal odour and colour of ginger, therefore, the fraud factor/indicator ‘Detectability of food fraud in final products’ is low vulnerability (Table 6 - Question 2 - Answer option 1 - score 1)^42^. This fraudulent behaviour was reported by the media, but it is less common in China recently, hence the ‘Historical evidence of fraud in final products’ is assigned a medium vulnerability level (Table 6 - Question 3 - Answer option 2 – score 2). Altogether, the frequency of all three factor/indicators is equal, no highest frequency and corresponding vulnerability can be determined at this stage.

#### Local collection

For the local collection stage (Stage II) of the supply network, the collectors purchase ginger from farmers and rarely process the ginger. At this stage, collectors do not process ginger, the factor/indicator ‘Availability of technology and knowledge to commit food fraud on final products’ is not applicable at this stage. However, unapproved/undeclared processing of ginger may already happen at the primary production stage (Stage I), and it is still easy to be noticed. Therefore, the factor/indicator ‘Detectability of food fraud in final products’ is low vulnerability (Table 6 – Question 2 – Answer option 1 – score 1). In addition, there is no information about the ‘Historical evidence’ at this stage. However, one common issue at this stage is the loss of traceability^43^. The main reason for this loss at the local collection stage might be due to the lack of traceability standards between farmers and collectors^44^. Paper-based systems are widely implemented for food traceability across the whole food industry^45^. While such systems are cheap they can lack accuracy in the recording and storage of data^44^. Robust digital systems for traceability are more expensive to implement, operate and maintain^46^. For smallholder ginger farmers who live in remote districts, it is difficult and costly to implement and apply such advanced systems for traceability. Based on the available information, the opportunities related vulnerability at this stage cannot be determined.

#### Local processing

Local processors obtain fresh ginger from local collectors on a regional basis. At Stage III, local processors may perform some basic processing, such as cleaning, sterilization packing and grinding or fine process into other form^47^. Adulteration may occur at this stage when ginger is ground into powder or processed into other forms^43^. Fraudsters may add inferior material to ground ginger to increase its weight or they may add unauthorized ingredients to enhance certain qualities of ginger for profit^29^. For instance, adding powdered beans to ground ginger to increase weight and adding Sudan dyes for a more vibrant colour^48,49^. Because simple/basic technologies and methods are available, and no specialist facilities are required, to adulterate the materials/products, the ‘Availability of technology and knowledge to commit food fraud on final products’ is high (Table 6 – Question 1 – Answer option 3 – score 3). In addition, from the previous investigation of the literature and food fraud databases, even though the fraud issues in ginger are not as common as in expensive spices, adulteration is the main issue that usually happened during ginger processing and there is a lack of robust techniques for detecting ginger adulteration. Hence, the factor/indicator ‘Detectability of food fraud in final products’ is high vulnerability and the factor/indicator ‘Historical evidence of fraud in final products’ is medium (Table 6 – Question 2 – Answer option 3 - score 3, Table 6 – Question 3 – Answer option 2 - score 2). To sum up, the opportunities related vulnerability at the local processing stage is high because the high vulnerability has the highest frequency.

#### Local market

There are four actor groups within the local market stage (Stage IV); the wholesaler, retailer, food manufacturer and food service operators. The common fraud issue at this stage is deliberate misrepresentation/mislabelling^36^. For ginger products, deliberate misrepresentation can be around the geographical origin or production system claims^50,51^. This fraudulent issue does not require advanced technologies, methods, facilities and/or knowledge because no additional processing is needed on ginger. Therefore, the factor/indicator ‘Availability of technology and knowledge to commit food fraud on final products’ is high vulnerability (Table 6 – Question 1 – Answer option 3 - score 3). Although some studies has been conducted to identify the misrepresentation/mislabelling issues in ginger products, those methods usually need advanced equipment and trained analysts are needed for its detection^52^. Moreover, such laboratory tests are usually expensive and time-consuming. Based on the description of ‘Detectability of food fraud in final products’, the vulnerability related to this factor/indicator is medium (Table 6 – Question 2 – Answer option 2 – score 2). According to the results of the literature and food fraud databases, there are limited cases/documentations (five statements), hence, the fraud factor/indicator ‘Historical evidence of fraud in final products’ is medium vulnerability (Table 6 – Question 3 – Answer option 2 - score 2). To that end, the opportunities relate vulnerability at this stage is medium because the medium vulnerability has the highest frequency.

#### International trade

At Stage VI, the ginger products from China are transported to the EU. Exporters and agents are working as an intermediary between the Chinese market and the EU market. According to the Guidance on Authenticity of Herbs and Spices^36^, the purchase of low-grade materials and the occurrence of mislabelling often happen at this stage in the herb and spice industry supply chain. The main fraud issue at this stage is the same as that at the local market stage. Consequently, the factor/indicators for this stage, including ‘Availability of technology and knowledge to commit food fraud on final products’, ‘Detectability of food fraud in final products’ and ‘Historical evidence of fraud in final products’ have the same vulnerability as the local market stage which were high, medium, and medium vulnerability, respectively. According to an interview with EU processors, to prevent food fraud, they only trade with exporters and agents who are trustworthy and have long-term partnerships. However, this cooperation model may lead to the EU processors being over-dependent on their exporters and agents. One of the interviewees from an EU spice company stated that all information about their purchase of ginger products came from their Chinese agents. Therefore, excessive reliance on agents or exporters creates fraud vulnerability at the international trade stage. To sum up, the opportunities related vulnerability at this stage is medium since the medium vulnerability has the highest frequency.

#### EU processing

The EU processors group of Stage VII is one of the most significant actor groups within the ginger supply chain network. Most of the ginger products in the EU market come from the EU processors. In addition to the basic processing such as cleaning, sterilization and grinding, the EU processors may further process ginger into other ginger products, for instance, pickled ginger, preserved ginger, and crystalized ginger depending on the companies. Like local processors in China, adulteration of ground ginger is still the main fraud issue at this stage, such as adding spent ginger to ground ginger^53^. Therefore, the EU processing stage has the same opportunities related vulnerability as the Local processing stage, which is high vulnerability. In addition, all interviewees at this stage claimed that they had confirmed the authenticity of their products and believed that the fraud issues they knew always came from other actor groups in the ginger supply chain. However, the questions related to food fraud may touch on the sensitivities of potential offenders, therefore, we presumed the interviewees might answer the questions in a more reserved or socially acceptable way. This behaviour is consistent with the ‘alien conspiracy theory’, which describes that crime as a problem from outside parties, rather than a part of the own direct environment/society^54^.

#### The EU market

Stage VIII consists of six actor groups that directly sell ginger products to EU consumers. At this stage, the fraud vulnerability would be knowingly placing mislabelled products on the market^43^. Therefore, the same in the local market, the opportunities related vulnerability at this stage is medium.

#### Consumers

At Stage V and Stage IX of the spice supply chain, local (Chinese) and EU consumers receive the final products circulating within the entire supply chain network. At these stages, it is likely that the products may already be tampered with if it has been vulnerable to food fraud at earlier stages of the network^55^.

#### The overall opportunities and motivations related vulnerability of the whole chain

##### The overall opportunities related vulnerability of the whole chain

**Fig. 2:**
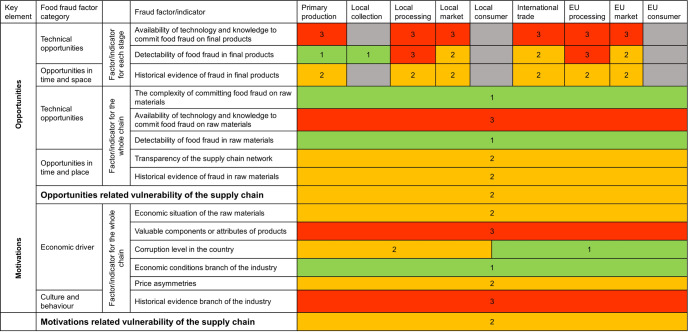
Visualization of the opportunities and motivations related vulnerability in ginger supply chain from China to the European Union (EU). Note: Number 1, 2 and 3 reflected a low, medium, and high vulnerability level.

The nine stages of the ginger supply chain from China to the EU and corresponding opportunities and motivations related vulnerability have been compiled in Fig. 2. The low, medium, and high vulnerability respectively corresponded to green, orange, and red colours for better visualization. The overall opportunities related vulnerability of the chain based on the results of nine stages is medium to high vulnerability because medium and high vulnerability have the same frequency. The local processing and EU processing have high opportunities related vulnerability compared with other stages. According to the theoretical framework of the FFVA tool, more robust internal hard controls should be applied at these two stages to counterweigh the high vulnerability in opportunities^1^.

In addition to the indicators targeting each stage, five additional Opportunities related indicators for the whole were also considered. The raw material (raw ginger) of the whole supply chain is consistent, therefore the factor/indicators related to raw material were considered based on the whole supply chain. For the factor/indicator ‘The complexity of committing food fraud on raw materials’, the physical status of raw ginger is in line with the description of Table 7 – Question 1 - Answer option 1 – score 1, which is low vulnerability. As discussed in the primary stage, the unapproved/undeclared processing of raw ginger does not require advanced technology/knowledge and can be easily noticed by odour and colour, therefore, the factor/indicator ‘Availability of technology and knowledge to commit food fraud raw materials’ is high vulnerability (Table 7 – Question 2 – Answer option 3 - score 3) and the factor/indicator ‘Detectability of food fraud in raw materials’ is low vulnerability (Table 7 – Question 3 – Answer option 1 - score 1). The factor/indicator ‘Historical evidence’ for the raw ginger is less common, consequently, this factor/indicator is medium vulnerability (Table 7 – Question 5 – Answer option 2 - score 2). For the factor/indicator ‘Transparency of the supply chain network’, the information from 13 relevant articles used to map the ginger supply chain indicated that the ginger supply chain is not fully transparent, there is a lack of research on the structure of the chain. Accordingly, the ‘Transparency of the supply chain network’ is medium vulnerability (Table 7 – Question 4 – Answer option 2 – score 2). To bring the factor/indicators targeting each stage and the indicator targeting the whole chain together, the overall opportunities related vulnerability of the whole ginger supply chain from China to the EU is medium since the medium vulnerability has the highest frequency.

##### The overall motivations related vulnerability of the whole chain

For the motivations-related vulnerability, all six motivations-related indicators are targeting the whole supply chain. Regarding the factor/indicator ‘Economic situation of the raw materials’, the ginger products fit the description of medium vulnerability (Table 7 – Question 6 – Answer option 2 – score 2) i.e., stable prices but the supply of raw materials are not readily available and export bans on raw materials exist in a few countries. The price of ginger is stable compared with expensive spices which have big price fluctuations, such as black pepper^56^. However, the export ban existing in a few countries makes ginger is not readily available all the time^57^. For the factor/indicator ‘Valuable components or attributes of products’, the ginger products fit the description of high vulnerability (Table 7 – Question 7 – Answer option 3 – score 3), i.e., the value of materials/products is greatly determined by its composition, way of production and/or origin. The factor/indicator ‘Corruption level in the country’ is low to medium vulnerability, since the corruption index is medium (66) for China and low for top import countries (Netherlands 8, Germany 10). The vulnerability of the factor/indicator ‘Economic conditions branch of the industry’ is low because the ginger industry is operating in a growing market according to Tridge (Table 7 – Question 9 – Answer option 1 – score 1). The information from Tridge also indicated that the factor/indicator ‘Price asymmetries’ is medium vulnerability since the price of ginger is different in some regions and countries (Table 7 – Question 10 – Answer option 2 – score 2). The last factor/indicator ‘Historical evidence branch of the industry’ was determined by fraud prevalence in ginger in previous section, there is well-known and documented evidence of fraudulent activity across the ginger supply chain. Therefore, the ‘Historical evidence branch of the industry’ is high vulnerability. In conclusion, the motivations related vulnerability of the ginger supply chain is medium vulnerability due to the high frequency of the medium vulnerability.

## Mitigating food fraud in the ginger supply chain

According to the Global Food Safety Initiative (GFSI) guidelines, two key elements are needed to mitigate the risk of food fraud, food fraud vulnerability assessment and control plan^58^. Until now there is no food fraud vulnerability assessment has been conducted on the ginger supply chain from China to the EU. In addition, based on the search results from the literature, as an key element of the control plan, laboratory-based detection techniques for ginger fraud issues are not well-developed. Limited research has been conducted on the detection of known adulterants in ginger products and the new emerging adulterants keep increasing the length of the adulterants list of ginger products. The development of robust detection techniques and the transfer from the laboratory to the real industry is still on the way. Therefore, the available food fraud vulnerability assessment tools and possible detection techniques will be discussed in this section.

### Potential vulnerability assessment tools for the industry

In the previous section, a modified food fraud vulnerability assessment tool (i.e., SSAFE FFVA) was applied to have a general impression of the food fraud vulnerability in the ginger supply chain. However, it is necessary to apply the fraud vulnerability assessment tools in the spice companies to reveal the real situation of the supply chain. Besides the SSAFE FFVA tool^59^ used in this study, various other food fraud vulnerability assessment tools have been developed to determine fraud risks of companies within supply chains. Examples include the vulnerability assessment and critical control points (VACCP), food fraud mitigation guidance^60^, CARVER plus shock method^61^, NSF fraud protection model^62^ and the food fraud initial screening model (FFIS)^63^. In principle, the above-mentioned food fraud vulnerability assessment tools do not provide specific mitigation techniques for ginger fraud, as indeed is the case for most commodities and ingredients but they provide the possibility to find vulnerable points along with the ginger supply chain network. However bespoke risk assessment tools are required as has been shown in the case of beef fraud^64^.

### Detection techniques for ginger fraud

In this study, the investigation in the literature indicated a lack of appropriate detection techniques for ginger fraud. The robust detection technique is one of the key elements in the control plan to mitigate food fraud. Therefore, in this section, ginger authentication in practice and potential detection techniques will be discussed to have a general impression on possible technical solution to ginger fraud.

#### Ginger authentication in practice

According to interviews with stakeholders, to ensure the authentication and safety of their ginger products, some technical control measures have been taken on the raw material and the final products of ginger. For the raw material, undeclared bleaching and SO_2_ addition can be detected using wet chemistry. In terms of detecting the adulteration of ginger products, especially in the ground form, there are several problems with the current control measures. An interviewee stated that traditional wet chemistry is costly for companies and DNA profiling for spice adulteration does not work properly. The public databases for DNA analysis are not valid and lack verification. In addition, some processing of ginger may destroy DNA making profiling inaccurate. Therefore, more accurate, low-cost, and efficient techniques need to be developed to address the real-life fraud issues in ginger.

Although studies on ginger authentication are limited, many techniques have been applied for the characterization of ginger (Table 8). Techniques used for characterization can also be used for authentication, because of the ability to distinguish the differences between groups of samples. Most of the characterization of ginger is based on the main active constituents in ginger, including volatile organic compounds and non-volatile pungent compounds which attribute to the unique flavour of ginger products^65–67^.

**Table 8:** Analytical techniques used for ginger characterization.

Aim of characterization	Techniques	Key compounds	Reference
The effects of different drying methods on the volatile components of ginger	HS-SPME-GC-MS	Zingiberene; β-phellandrene; β-sesquiphellandrene; geranial	^76^
Identification of volatile constituents in ginger	GC–MS	54 compounds including geranial, zingiberene, β-sesquiphellandrene and β-phellandrene	^77^
Analysis of the volatile compounds associated with pickling of ginger	GC-IMS	Heptanal, heptanone, butanal, butanone, methional	^78^
Flavour changes in ginger during microwave vacuum drying	LF-NMR	51 volatile compounds including alkenes, esters, alcohols, aldehydes, and ketone	^23^
Identification of ginger volatiles and localization of aroma-active constituents	GC–Olfactometry	Gerania, eucalyptol, β-linalool, bornyl acetate	^79^
Analysis of volatile and non-volatile compositions in ginger oleoresin	GC–MS	Volatile compounds: alpha-zingiberene, beta-sesquiphellandrene, alpha-farnesene, beta-bisabolene, alpha-curcumene. Pungent compounds: 6-gingerol, 6-shogaol, zingerone	^80^
Characterization of cultivars of ginger	HPTLC; HPLC	6-gingerol, 8-gingerol, 10-gingerol, 6-shogaol	^30^
Discrimination of ginger according to geographical origin	HPLC-DAD	Gingerols and other gingerol-related compounds	^66^
Discrimination of ginger according to geographical origin	Label-free proteomic analysis	Proteins	^81^
Discrimination of ginger varieties	CLC	Gingerols, shogaol	^32^
Profiling of phenolic composition in normal ginger and black gingers	UPLC–DAD–QToF–MS	Phenolic composition	^82^
Characterization of ground ginger with different particle sizes	XPS; SEM	-	^83^

*CLC* Capillary liquid chromatography, *GC-IMS* Headspace gas chromatography‐ion mobility spectrometry, *GC*–*MS* Gas chromatography–mass spectrometry, *GC*–Olfactometry Gas chromatography–olfactometry–mass spectrometry, *HPLC* High performance liquid chromatography, *HPLC*-*DAD* High-performance liquid chromatography with a diode-array detector, *HPTLC* High performance thin layer chromatography, *HS*-*SPME*-*GC*-*MS* Headspace solid-phase microextraction followed by gas chromatography–mass spectrometry, *LF*-*NMR* Low-field nuclear magnetic resonance, *SEM* scanning electron microscope, UPLC–DAD–QToF–MS Ultra performance liquid chromatography coupled with diode array detector, quadrupole time-of-flight mass spectrometry, *XPS* X-ray photoelectron spectroscopy.

#### Potential ginger authentication techniques

Previous studies on ginger characterization provide the possibility to explore different analytical techniques to detect ginger fraud. In addition, the limitation of current methods for ginger authentication in practice creates a need to develop better techniques. According to a database of food ingredient fraud and economically motivated adulteration generated by Moore et al. (2012), chromatography, vibrational spectroscopy, mass spectrometry and DNA-based analyses were the most common approaches applied. These analytical tools can be further divided into confirmatory methods and screening techniques. The principle of confirmatory techniques for spice adulteration is mainly based on the identification of specific markers of the spice, such as adulterants and specific chemical constituents (Galvin-King et al., 2018). The application of screening techniques for spice adulteration are characterized as being rapid, low cost and non-destructive. Both confirmatory and screening techniques could be applied to detect adulteration in ginger products.

Based on the principle of confirmatory techniques, certain adulterants in ginger products and some characteristic compounds in ginger can be used as specific markers in detecting ginger fraud. For the adulteration of processed ginger products, illegal dyes, unauthorized preservatives, and undeclared sweeteners are the most used adulterants. Researchers have demonstrated that these adulterants can successfully be detected by some techniques, such as high-performance thin-layer chromatography-mass spectrometry and thin layer chromatography^68,69^. These confirmatory techniques are characterised as being more labour intensive, costly, and accurate as opposed to screening techniques.

Screening techniques can be powerful tools for non-targeted analysis to detect ginger adulteration. In practice, they are applied to flag suspect products which can then be further investigated with the more accurate confirmatory methods. For ground ginger, like many spices, the list of adulterants could be endless^70^. Some non-targeted screening methods are more suitable for rapid quality testing. For instance, near-infrared (NIR) spectroscopy has been used to distinguish pure black pepper powder from adulterated pepper samples mixed with black pepper husk, papaya seeds, pinheads and chili powder^71^. Although still in the early stages of development and practical application, the advantages of non-targeted screening techniques on food fraud will make them one of the most suitable tools in combating ginger fraud.

## General discussion of the overall ginger supply chain

One of the main aims of this review was to summarize the open access data about ginger fraud from the food fraud databases and the published literature. A second aim was to map the structure of the ginger supply network from China to the EU to identify the most vulnerable nodes along the chain and to propose analytical approaches that can be taken to detect and help mitigate fraud. Figure 2 from the Section: The ginger supply chain network: from China to the EU indicated that the overall opportunities and motivations related vulnerability of the ginger supply chain from China to the EU is medium vulnerability. The figure also indicated a difference in the vulnerability at different stages. To prevent food fraud, technical control measures should be applied at different stages. The undeclared/unapproved processing issue at the primary production stage can be detected very easily by the observation of odors and colour and wet chemistry. For monitoring the local market, international trade and EU market stages, isotope ratio mass spectrometry and elemental analysis can be used as an effective authentication technique to detect misrepresentation/mislabeling of geographical origin/production system claims despite the high cost^72,73^. For the adulteration of ground ginger and processed ginger at local processing and EU processing stages, the various potential techniques mentioned in the section: Potential ginger authentication techniques can be the analytical solutions in the future. According to the statements of the stakeholders, the current analytical control measures do not meet their expectations (as described in the section: Ginger authentication in practice). Therefore, the development of more promising and less costly analytical techniques is needed.

As a typical low-priced and high trade volume spice product, this is the study to investigate the food fraud prevalence in ginger products from the perspective of the supply chain. However, certain limitations of this study should be considered. First, there were only five participants for the stakeholder interviews, limiting the information from stakeholders and representing their perspectives. Second, only English literature and food fraud databases were used in this study. This could cause the omission of the food fraud information reported in other languages. Third, only open access information in the food fraud databases was included in this study. Despite these limitations, the current results still provide a good understanding of food fraud in the supply chain of ginger products and provide possible analytical solutions for detecting food fraud in the ginger supply chain from China to the EU.

## Conclusion

Food fraud is a constant threat to the spice industry. The results of the food fraud databases and the literature indicate that ginger fraud is a threat that has not been well investigated by researchers, especially the adulteration issues of ground ginger. Even though adulteration in ginger was widely reported in the literature, the available detection techniques, specifically focussed on ginger fraud, are limited. In addition, by mapping the ginger supply chain from a fraudulent perspective, the opportunities and motivation related vulnerability of the ginger supply chain was considered to be at a medium with the processing stages identified as the most vulnerable nodes along the chain. Confirmatory and screening techniques based on ginger characteristics analysis should be applied and developed in the future. In summary, attention should be given not only to extremely high valuable spice products, but also to spice products marketed at lower prices and sold in high trade volumes.

## Supplementary information

Supplementary Material

## Data Availability

The authors declare that all data supporting the findings of this study are available within the article and its supplementary information files.
