# Supplementary material for: A sense of ginger fraud: prevalence and deconstruction of the China-European union supply chain
Source: NPJ Sci Food. 2022 Nov 4;6:51. doi: 10.1038/s41538-022-00166-y (PMC9633793; doi:10.1038/s41538-022-00166-y)
Supplement: Supplementary file 1 — Supplementary Material [file 41538_2022_166_MOESM1_ESM.pdf]

Supplementary Table 1 Databases for collecting ginger and ginger fraud cases.

Database	Description	Uniform Resource Locator (URL)
Food And Feed Safety Alerts Portal (RASFF Portal)	The RASFF Portal records new cases of health risks found in food or feed.	https://ec.europa.eu/food/safety/rasff/portal_en
Food Fraud Risk Information	Food Fraud Risk Information contains food fraud risk information about hundreds of different food types, including past incidences of food fraud and emerging threats.	https://trello.com/b/aoFO1UEf/food-fraud-risk-information
Decernis Food Fraud Database	Decernis Food Fraud Database is a continuously updated collection of thousands of ingredients and related records gathered from scientific literature, media publications, regulatory reports, judicial records, and trade associations from around the world.	https://decernis.com/
Food Adulteration Incidents Registry	Information for this database is collected through literature and media, involving economically motivated and intentional adulteration incidents in food products.	https://foodprotection.umn.edu/fair
Recalls, Market Withdrawals, & Safety Alerts	This database provides information about certain recalls of FDA-regulated products.	https://www.fda.gov/safety/recalls-market-withdrawals-safety-alerts
Medical Information System (MEDISYS)	MEDISYS provides a monitoring system to identify	https://medisys.newsbrief.eu/medisys/homeedition/en/home.html

Database	Description	Uniform Resource Locator (URL)
	possible public threats rapidly.	
Food and Agriculture Organization Corporate Statistical Database (FAOSTAT)	The data in FAOSTAT includes the production, price, trade, etc. of agricultural plants.	http://www.fao.org/faostat/en/#data
Tridge	Tridge is a website that reports the market overview of certain products. Production, import and export information can be found in Tridge.	https://www.tridge.com/

Supplementary Table 2 Publications related to spice or ginger supply chain network.

No.	References
1	(FAO, 2019)
2	(Centre for the Promotion of Imports from developing countries, 2019)
3	(CBI Ministry of Foreign Affairs, 2015)
4	(Dessie et al., 2019)
5	(Lakner et al., 2018)
6	(Galvin-King et al., 2018)
7	(Thanushree et al., 2019)
8	(Schaarschmidt et al., 2018)
9	(Sharangi, 2018)
10	(Vodenicharova, 2020)
11	(Queensland Government, 2009)
12	(BRC-FDF-SSA, 2016)
13	(Herms, 2015)

Supplementary Table 3 Interview questions for the stakeholders in the spice supply chain.

General questions:

Name of the company:

Country:

Name of the contact person:

Position of the contact person:

Specific questions:

1. What is the function of your company within the spice industry (i.e., producer, trader, supplier)?

2. What does your general spice supply chain look like (behind and ahead of your company)?

3. Does this supply chain vary for different spices (i.e., pepper, turmeric, ginger)?

4. If so (Question 3), can you please provide an example of how the general supply chain can vary for different spices?

5. Does this supply chain vary for different forms of the different spices (i.e., fresh vs. whole/dried vs. ground/dried)?

6. If so (Question 5), can you please provide an example of how the general supply chain can vary for different forms of the different spices?

7. Does your company handle different ginger products? If so, what type of ginger products are received?

8. Does the supply chain(s) differ for these ginger products?

9. If the ginger product(s) (mentioned in Question 7) is processed, what kind of processing, where in the supply chain and/or by whom is the ginger product(s) processed?

Ginger product: [Name of the product as mentioned in Question 7]

Processing performed: [Kind of processing performed]

Node in the supply chain: [Position in the supply chain where the processing is performed]

Stakeholder(s) involved: [Stakeholder who performs the processing]

10. Are you aware of food fraud within the spice industry?

11. Have you specifically heard about food fraud with ginger in the past three years?

13. What is the impact (if any) of fraud on the spice supply chain?

14. Are there any kinds of control measures in your company to guarantee the authentication of your ginger products?

Supplementary Table 4 The detailed information about ginger fraud in the food fraud databases exhibited in Table 2.

Databases	Product	Fraud type	Detailed issue	Notifying countries
RASFF Portal	Pickled ginger	Adulteration	Too high content of sweetener	Poland
	Sliced ginger	Adulteration	Undeclared preservative	Denmark
	Sliced ginger	Adulteration	Undeclared preservative	Denmark
	Pickled ginger	Unapproved/undeclared processing	Undeclared colour	Poland
	Candied ginger in syrup	Adulteration	Undeclared preservative	Denmark
	Pickled ginger	Adulteration	Undeclared sweetener	Denmark
	Pickled ginger	Unapproved/undeclared processing	Undeclared colour	Spain
	Minced ginger	Adulteration	Undeclared preservative	Finland

Databases	Product	Fraud type	Detailed issue	Notifying countries
	Pickled ginger	Unapproved/undeclared processing	Undeclared colour	Cyprus
	Dried ginger	Adulteration	Undeclared sweetener	Lithuania
	Sliced ginger pickles	Unapproved/undeclared processing	Undeclared colour	Finland
	Picked pink ginger	Adulteration	Undeclared sweetener Undeclared preservative	Germany
	Pink sushi ginger	Unapproved/undeclared processing	Undeclared colour	Poland
	Candied ginger	Adulteration	Undeclared preservative	Denmark
	Ginger paste	Adulteration	Undeclared preservative	United Kingdom
	Ginger paste	Adulteration	Undeclared preservative	United Kingdom

Databases	Product	Fraud type	Detailed issue	Notifying countries
	Preserved ginger	Adulteration	Undeclared preservative	Denmark
	Pickled ginger	Unapproved/undeclared processing	Undeclared colour	Denmark
	Pickled ginger	Unapproved/undeclared processing	Undeclared colour	Cyprus
	Pickled ginger	Unapproved/undeclared processing	Undeclared colour	Denmark
Decernis Food Fraud Database	Ground ginger	Adulteration	Bean powder	
	Dried ginger	Adulteration	Ultramarine blue	
	Ground ginger	Adulteration	Lead	
	Ginger paste	Adulteration	Mashed potatoes; Acid (unspecified); Onions; Colour (unspecified); Banana pulp	India

Databases	Product	Fraud type	Detailed issue	Notifying countries
	Ginger	Adulteration	Sulfur dioxide; Calcium hypochlorite	India
	Ground ginger	Adulteration	Colour filler	
	Ginger paste	Adulteration	Synthetic substances	India
	Ginger oil	Adulteration	Soybean oil	
	Ginger	Misrepresentation of production system claims		
	Ginger	Misrepresentation of production system claims		
Food fraud Risk Information	Ginger	Adulteration/Misrepresentation of geographical origin		

Supplementary Table 5 The references of the articles in Table 3.

Key words	Number of the articles	References
Web of science		
"ginger" AND "fraud"	None	n.a.
"ginger" AND "adulteration"	6	(Danciu et al., 2018; Fasoli & Righetti, 2015; Ghosh et al., 2011; Mannina et al., 2012; Osisiogu, 1973; Sudershan, R. V., & Bhat, 1995)
"ginger" AND "unapproved processing" OR "undeclared processing"	1	(Wu et al., 2018)
"ginger" AND "mislabeling" OR "misrepresentation"	None	n.a.
Scope		
"ginger" AND "fraud"	1	(Mosa et al., 2018)
"ginger" AND "adulteration"	Overlapped with Web of science	
"ginger" AND "unapproved processing" OR "undeclared processing"	Overlapped with Web of science	

Key words	Number of the articles	References
“ginger” AND “mislabeling” OR “misrepresentation”	Overlapped with Web of science	
<u>Google scholar</u>		
“ginger” AND “fraud”	None	n.a.
“ginger” AND “adulteration”	21	(Parvathy et al., 2014)(Salmon et al., 2012)(Ali et al., 2008)(Galvin-King et al., 2020)(Rafi et al., 2013)(Mbogning Feudjio et al., 2017)(Jiang et al., 2006)(Allen, 1894)(Jelled et al., 2015)(Nair, 2013)(Rahman et al., 2015)(Ghosh, A. K., Banerjee, S., Mullick, H. I., & Banerjee, 2011)(Galvin-King et al., 2019)(Zhu HongMei & Zhao Meng, 2014)(Alanamu ABDULRAHAMAN, A., Oyeleye TAIWO, M., & Ayotunde, 2015)(Mohiuddin, 2019)(Subba Rao et al., 2007)(Oliveira et al., 2019)(Ercioglu et al., 2018)(Parveen et al., 2019)(Prashant Neupane, 2020)
“ginger” AND “unapproved processing” OR “undeclared processing”	None	n.a.
“ginger” AND “mislabeling” OR “misrepresentation”	2	(Rafi et al., 2013)(Nair, 2013)

Note. (n.a.) Not applicable

Supplementary Table 6 Not applicable fraud factor/indicators.

Elements	Fraud factor category	Fraud factor/indicator	Questions
Opportunities	Opportunities in time and place	Easiness interference processing lines	How would you describe the production lines/processing activities of your company?
Motivations	Economic drivers	Economic conditions own company	How would you describe the economic condition of your company?
		Companies establish prices in collaboration with suppliers (based on supplier profit data) and in agreement with common (worldwide) market prices	How would you describe the financial strains imposed by your company on your direct supplier(s)?
		Level of competition branch of industry	How would you rate the level of competition across your sector of the food supply chain (i.e., your company and your direct competitors)?
		Economic conditions supplier	How would you describe the economic health of your direct supplier(s)?
	Culture and behaviour	Organizational strategy own company	What are the characteristics of the business strategy of your company?

Elements	Fraud factor category	Fraud factor/indicator	Questions
		Ethical business culture own company	How would you describe the ethical business culture of your company?
		Criminal offences own company	Has your company been involved in criminal offences previously?
		Organizational strategy supplier	What are the characteristics of the business strategy of your direct supplier(s)?
		Ethical business culture supplier	How would you describe the ethical business culture of your direct supplier(s)?
		Criminal offences supplier	Has your direct supplier(s) been involved in criminal offences previously?
		Victimization of supplier	Has your direct supplier(s) been a victim of food fraud committed by their suppliers, customers, or other parties?
		Criminal offences supplier	Has your customer(s) been involved in criminal offences previously?

Elements	Fraud factor category	Fraud factor/indicator	Questions
		Ethical business culture branch of industry	How would you describe the ethical business culture across your sector of the food supply chain (i.e., your company and your direct competitors)?
Control measures	Technical measures	Fraud monitoring system raw materials	How would you rate your company's raw material monitoring control systems' ability to detect fraud?
		Verification of fraud monitoring system raw materials	Are the fraud monitoring tasks of your raw material control system verified in your company?
		Fraud monitoring system final product	How would you describe the fraud related parts of your final product monitoring control system of your company?
		Verification of fraud monitoring system final product	Are the fraud monitoring tasks of your final product control system verified in your company?
		Information system own company	How extensive is the information system for internal control of mass balance flows in your company?
		Tracking and tracing system own company	How extensive is the tracking & tracing system of your company?

Elements	Fraud factor category	Fraud factor/indicator	Questions
		Mass balance flow control system supplier	How extensive is the information system for control of mass balance flows of your direct supplier(s)?
		Tracking and tracing system supplier	How extensive is the traceability system of your direct supplier(s)?
		Fraud contingency plan	Does your company have fraud contingency measures in place?
	Managerial measures	Integrity screening employees own company	Is integrity screening of employees common procedure in your company?
		Ethical code of conduct own company	Is there an ethical code of conduct or guideline in place and embedded in your company?
		Whistle blowing own company	Is there a whistle blowing system (system for reporting assumed fraudulent activities) in place in your company?
		Contractual requirements suppliers	Do contractual requirements with your direct suppliers include elements that limit opportunities for fraud?

Elements	Fraud factor category	Fraud factor/indicator	Questions
		Fraud control system supplier	What features the fraud control system of your direct supplier(s)?
		Social control chain network	How would you describe the social control and transparency of actions across your supply chain?
		Fraud control system supplier	How well established is guidance for fraud prevention and control across your sector of the food supply chain? (i.e., your company and your direct competitors)
		National food policy	How would you describe your national food policy? (i.e., country-level)
		Enforcement practice chain network	How well are fraud prevention laws enforced locally?
		Enforcement practice chain network	How well are fraud related laws enforced across your international supply chain?
